# Addressing family communication in genetic counseling: A scoping review of process studies

**DOI:** 10.1002/jgc4.70067

**Published:** 2025-08-13

**Authors:** Maria Barbosa, Milena Paneque, Sofia Fontoura Dias, Filipa Júlio, Jorge Sequeiros, Liliana Sousa, Angus Clarke, Alison Metcalfe, Célia M. D. Sales, Álvaro Mendes

**Affiliations:** ^1^ i3S – Instituto de Investigação e Inovação Em Saúde University of Porto Porto Portugal; ^2^ CGPP – The Centre for Predictive and Preventive Genetics, IBMC – Institute for Molecular and Cell Biology University of Porto Porto Portugal; ^3^ FPCEUP ‐ Faculty of Psychology and Educational Sciences University of Porto Porto Portugal; ^4^ CPUP ‐ Center for Psychology University of Porto Porto Portugal; ^5^ ICBAS ‐ School of Medicine and Biomedical Sciences University of Porto Porto Portugal; ^6^ Department of Education and Psychology University of Aveiro Aveiro Portugal; ^7^ RISE‐Health, Health Research and Innovation University of Aveiro Aveiro Portugal; ^8^ European Huntington Association Moerbeke‐Waas Belgium; ^9^ Portuguese Huntington Association Lisbon Portugal; ^10^ Faculty of Psychology and Educational Sciences University of Coimbra Coimbra Portugal; ^11^ CIBIT – Coimbra Institute for Biomedical Imaging and Translational Research University of Coimbra Coimbra Portugal; ^12^ Cardiff University School of Medicine, The Sir Geraint Evans Building University Hospital of Wales Cardiff UK; ^13^ LOHA Health Ltd London UK; ^14^ Department of Diagnostics and Intervention, Oncology Umeå University Umeå Sweden

**Keywords:** family disclosure, genetic counseling, genetic risk, literature review, psychosocial genetics

## Abstract

Process studies explore the content and dynamics established during genetic counseling (GC), allowing a greater understanding of what happens. No literature review has specifically examined how family communication of genetic information has been addressed in GC process studies. To fill this gap, a scoping review was conducted. Scopus, Web of Science, PubMed, and PsycInfo were searched, resulting in 21 articles for analysis. Most studies were retrospective (*n* = 19) and qualitative (*n* = 15) and involved hereditary cancer syndromes (*n =* 13). Studies analyzed how family communication of genetic information is addressed in GC by either focusing on patients' experiences and perspectives, or genetic healthcare professionals' (GHP) roles and scope of practice. All studies reported that GHP address family communication with patients, but their practices were heterogeneous and influenced by contextual factors. Practices to address family communication included providing guidance to inform the family (*n* = 19), materials to support communication (*n* = 16), psychosocial assessment (*n* = 11), and additional support (*n* = 18). Our findings suggest that the approach to family communication in GC draws on both teaching and counseling models, although with greater emphasis on the former. This is consistent with integrated models of GC. Future prospective process studies using observational data could enhance our understanding of patient‐professional interactions and their influence on patient decision‐making regarding family communication of genetic information.

**What is known about this topic**

- Guidelines recommend genetic healthcare professionals (GHP) encourage and support patients in sharing relevant information with at‐risk relatives.
- Process studies may help clarify how communication with the family is addressed in genetic counseling (GC). However, no reviews have specifically focused on GC process studies addressing communication with relatives.

**What this paper adds to the topic**

- This review summarizes existing process studies on GC that address family communication, showing that research is mostly qualitative and retrospective.
- GHP consistently inform patients of their relatives' genetic risk, the importance of family communication, provide written resources and additional support to assist with informing relatives. However, GHP less frequently assess patients' ability to do so and tailor their support.

## 1 INTRODUCTION

Genetic and genomic testing are becoming increasingly accessible and comprehensive, and thus are more frequently being mainstreamed into other specialized areas of healthcare beyond genetics (McNeill, 2022). The implications of test results often extend to patients' biological relatives, who might be at risk of developing or passing on genetic conditions to their offspring.

Informing relatives of their risk enables them to make informed decisions on their health and reproduction, as well as access genetic counseling (GC) and cascade screening. Relatives with a pathogenic variant can access surveillance, prevention or treatments (when available) that can mitigate future morbidity, as well as reproductive options (Strachan & Lucassen, 2022). Families' adjustment to this information is often punctuated by relational, behavioral, and emotional implications. Patients and their families often experience relief, distress, anxiety, guilt, or a sense of empowerment during this process (Elrick et al., 2017; Gaff et al., 2007; Mendes et al., 2018). Knowledge of a genetic condition in the family system might also entail various relatives making decisions about testing and changing plans regarding health, reproduction and other aspects of life. This might translate into adjusting family roles, communication patterns, and support dynamics (Ahsan et al., 2023; Bowen et al., 2021; Chivers Seymour et al., 2010; Fontoura Dias et al., 2025; Forrest et al., 2003; Gaff et al., 2007; Gomes et al., 2022; Mendes et al., 2018; Shah & Daack‐Hirsch, 2018; Wiseman et al., 2010; Young, Butow, Rhodes, et al., 2019).

Commonly, patients are the ones informing their relatives about hereditary conditions and genetic risk. While most understand the importance of sharing this information and intend to do so (Finlay et al., 2008; Hunter et al., 2023), many find it difficult. How individuals communicate this information to relatives is influenced by disease characteristics and individual, relational, and social influences. This includes family dynamics, communication patterns, perceived importance of the information, anticipated reactions from relatives, and the psychological adjustment to one's test results (Chivers Seymour et al., 2010; Fontoura Dias et al., 2025; Forrest et al., 2003; Gaff et al., 2007; Gomes et al., 2022; Wiseman et al., 2010; Young, Butow, Rhodes, et al., 2019). As such, many would appreciate more support from genetic healthcare professionals (GHP) (Marleen van den Heuvel et al., 2020).

GC aims to support consultands in understanding and adapting to the implications of genetic conditions, including familial implications and facilitating its effective communication within families (Resta et al., 2006). Guidelines generally advise GHP to encourage and support patients in sharing relevant information with at‐risk relatives (Phillips et al., 2021). Two approaches are typically used: (i) in family‐mediated contact, the default practice, information is relayed to relatives through the proband; (ii) in direct contact, GHP directly contact relatives (Mendes & Newson, 2024).

In family‐mediated contact, the patient informs at‐risk relatives of their potential risk and availability of GC, without necessarily disclosing their own genetic status or test results (carrier, noncarrier or inconclusive). Carriers frequently find it easier to share this information with their families, as it promotes communication and awareness of familial risk and testing (Cirino et al., 2022; Patch & Middleton, 2018). Those who disclose genetic information often do so out of responsibility toward relatives and to obtain further information and support (Afaya et al., 2024; Gaff et al., 2007; Gomes et al., 2022; Greenberg & Smith, 2016). However, patients might also want to withhold disclosure to protect themselves and relatives from distress and potential discrimination (Afaya et al., 2024; Fontoura Dias et al., 2025; Hunter et al., 2023; Mendes et al., 2018). Conversely, in the communication of noncarrier or inconclusive test results patients might experience guilt and/or emotional distress (Mendes et al., 2018), and relatives might have difficulties or incorrectly interpret their own risk (Afaya et al., 2024; Himes et al., 2019).

Few studies have examined how family communication is addressed in GC. A systematic review suggested that GHP encourage family communication, provide psychoeducational guidance, and written information to distribute among at‐risk relatives (Mendes et al., 2016). This review did not explore the perspectives of patients and their families about how communicating genetic information with at‐risk relatives is addressed in GC. It also excluded studies published after 2014 (Mendes et al., 2016), thereby not considering the potential changes in service provision since then. Notably, this includes increased demand and accessibility to GC and genetic testing (Gima et al., 2024; Ormond et al., 2024; Wallgren et al., 2021; Zakaria et al., 2023), updates to practice guidelines and policies (Phillips et al., 2021), increased integration of digital tools and telehealth into GC, and a growing emphasis on considering context (cultural, social, and economic factors) to tailor communication strategies and support (Biesecker, 2020; Ormond et al., 2024; Shete et al., 2024; Wallgren et al., 2021; Zakaria et al., 2023). Other reviews examined guidelines for addressing family communication (Forrest et al., 2007; Phillips et al., 2021) and the impact of interventions on the rate of at‐risk relatives seeking GC and testing (Ballard et al., 2023; Baroutsou et al., 2021; Law et al., 2022; Young et al., 2023; Zhao et al., 2022). However, these provide limited insight into how family communication is approached by GHP during GC.

GC practice has been described as a “black box” (Biesecker & Peters, 2001), and the need for further research into the process of GC has been emphasized to better evaluate service models and understand their outcomes (Biesecker & Peters, 2001; Clarke et al., 1996). Process studies aim to investigate the content, behaviors, and relationships established during GC (Biesecker & Peters, 2001). Unlike other types of research, they extend beyond assessing outcomes and examine real‐world clinical practice, thereby illuminating the interactions, challenges and opportunities within the GHP–patient encounter (Biesecker, 2020; Biesecker & Peters, 2001; Clarke et al., 1996; Meiser et al., 2008; Paul et al., 2015; Roter et al., 2006). In doing so, they may provide practice‐based evidence that can help inform the development of practice guidelines. Process studies have been central to defining GC, including its goals, models, interventions, and GHP's scope of practice (Aasen & Skolbekken, 2014; Biesecker, 2020; Biesecker et al., 2021; Boghosian et al., 2021; Guerra et al., 2023; Kessler, 1997; MacLeod et al., 2018; Meiser et al., 2008; Redlinger‐Grosse et al., 2017; Schmidlen et al., 2018; Veach et al., 2007). When these studies explore communication, they usually examine the style and content of interactions between patients and GHP (Aasen & Skolbekken, 2014; Joseph et al., 2017; Roter et al., 2006; Scott et al., 2024). When they focus patients' experiences, commonly they report their needs, expectations, attitudes, awareness, and recommendations (Dwyer et al., 2022; Hodan et al., 2024; Pollard et al., 2020; Rolle et al., 2022).

Process studies that explore GHP's, patients', and their relatives' perspectives may help clarify how family communication of genetic information is addressed in GC. However, to date, no reviews have specifically focused on process studies exploring this, which may limit our understanding of GC practice, and the support provided to patients in this context. This scoping review extends the prior work of Mendes et al. (2016) by including more recent studies to reflect contemporary shifts in genomic medicine, and examining both GHP, patients' and relatives' perspectives on how family communication of genetic information is addressed in GC. Therefore, this review aims to map the available GC process research, focusing on (i) which methodological approaches have been used to study family communication of genetic information, (ii) what dimensions of the GC process have been focused on, and (iii) how GHP address family communication during GC.

## 2 METHODS

A scoping review was conducted following the Preferred Reporting Items for Systematic Reviews and Meta‐Analyses (PRISMA) (Tricco et al., 2018) and Joanna Briggs Institute (JBI) guidelines (Aromataris et al., 2024; Peters et al., 2021) (Table S1). The protocol was registered with Open Science Framework (https://doi.org/10.17605/OSF.IO/MB2AX).

### 2.1 Eligibility criteria

The Population, Concept, Context approach was used to define inclusion and exclusion criteria (Table 1). The selected sources of evidence included peer‐reviewed primary research published in Portuguese, Spanish, French, or English since 1997, when direct mutation detection and the widespread adoption of presymptomatic testing became available (Tibben, 2007) to 2023.

**TABLE 1:** Eligibility criteria.

Inclusion	Exclusion
Population	
GHP conducting GC consultations Individuals undergoing or having received GC	GHP not engaged in GC Individuals who have not received GC
Concept	
Process studies examining how family communication of genetic information is addressed in GC from the perspective of (i) GHP (strategies and actions used or that could be used to address family communication) or (ii) patients (perceptions of how family communication was addressed in GC, and suggestions of practices to explore family communication)	Process studies that do not cover (i) how family communication of genetic information is approached in GC or (ii) the interactions between GHP and patients regarding family communication Studies that solely focus on family communication as an outcome of GC, without describing how it is addressed in practice
Context	
GC research that explores the communication of genetic information to the family	Other contexts that address family communication of genetic information

### 2.2 Search strategy

An initial search of PubMed was undertaken to identify relevant articles. Query strings were defined based on their titles, abstracts, and index terms. These were used to develop the search strategy for PsycINFO‐EBSCOhost, PubMed, Scopus, and Web of Science (Table S2) during July 2023 (the final database search was conducted on 8 August 2023). This search was supplemented by manual citation searching in the reference lists of included articles (conducted between 9 and 14 August 2023).

Identified articles were imported into EndNote 21 (Clarivate Analytics, PA, USA) and duplicates were removed. A first screening of titles and abstracts using Rayyan (Qatar Computing Research Institute, Doha, Qatar), and full‐text screening was then conducted independently by MB and SFD. Disagreements (*n =* 4) were resolved by discussing with ÁM if the articles were eligibility until an agreement was reached.

### 2.3 Data extraction

MB conducted the data extraction and SFD assessed its completeness and accuracy. The extraction table (Table S3) was modified iteratively throughout the extraction based on input from ÁM, MP, and CS. Extracted data included author, year, country, title, study aim(s), author's background, genetic conditions, participants, sample size, field of the healthcare professionals, study methodology, dimensions of GC, practices to address family communication, influencing contextual factors, patient‐suggested practices and GHP's resource needs.

### 2.4 Data analysis

The extracted data were analyzed through descriptive statistical analysis and iterative content analysis (Peters et al., 2021; Pollock et al., 2023). This entailed familiarization with the data, initial open coding, and creating an inductive coding framework to categorize extracted data based on the research questions (Pollock et al., 2023). Findings were presented with figures, tables, and a narrative summary (Pollock et al., 2023). Per the established guidelines (Aromataris et al., 2024; Peters et al., 2021; Tricco et al., 2018), a quality rating of the studies was not conducted.

## 3 RESULTS

**FIGURE 1:**
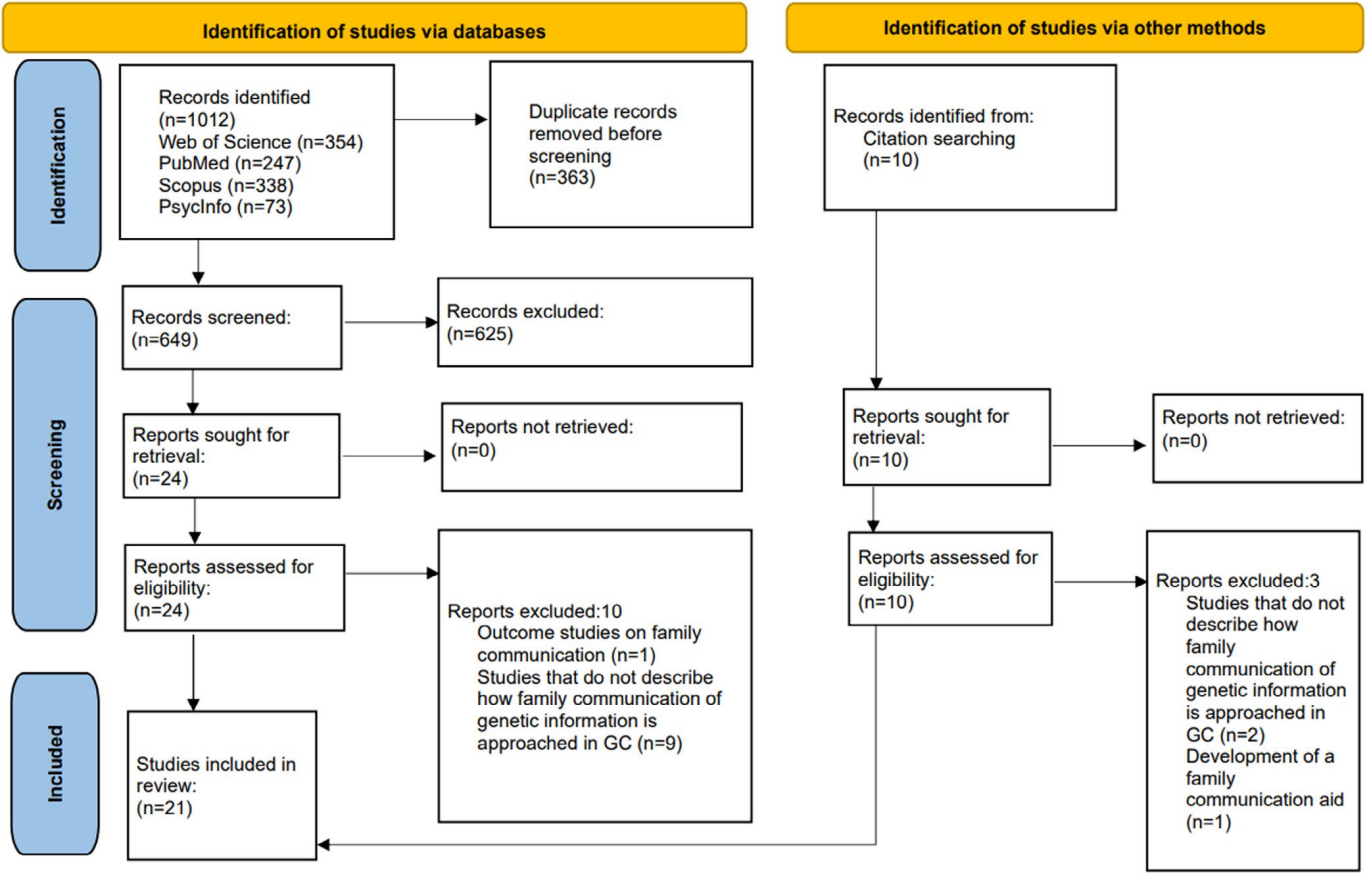
PRISMA flowchart outlining the database and citation searching process.

A total of 1012 potential articles were identified. After removing duplicates (*n =* 363), the title and abstract of 649 articles were screened, of which 24 underwent full‐text review. Ten additional articles were identified through reference and citation searching. Twenty‐one articles met the eligibility criteria and were included in the review (Figure 1).

### 3.1 Overview of study characteristics and methods

Table S4 summarizes the characteristics of the included studies. These were published from 2005 to 2022, with 14 (67%) published between 2016 and 2022. Most studies were conducted in Australia (*n =* 6; 29%) and the United States of America (*n =* 5; 24%). Most studies (*n =* 11; 52%) described multiple genetic conditions, with hereditary cancer syndromes being the most frequent (*n =* 13; 62%), particularly breast and ovarian cancer (*n =* 9; 43%).

In 15 studies (71%), multidisciplinary care teams were involved in GC. GHP were the most frequently mentioned, with 16 studies mentioning genetic counselors (76%) and 10 mentioning medical geneticists (48%).

Most studies (*n =* 16; 76%) were conducted by multidisciplinary research teams. Researchers most often had a background in GC (*n =* 14; 67%) or clinical genetics (*n =* 11; 52%). Reviewed studies were predominantly qualitative (*n =* 15; 71%), retrospective (*n =* 19; 90%), and five (24%) reported both process and outcomes of GC relating to family communication. Most employed a single method for data collection (*n =* 15; 71%) and analysis (*n =* 16; 76%), with semi‐structured interviews (*n =* 9; 43%) and thematic analysis being the most common (*n =* 6; 29%; Table 2).

**TABLE 2:** Methodological approaches of reviewed studies.

Methodological approach	*n*	%
Qualitative	15	71
Mixed methods	5	24
Quantitative	1	5
Retrospective	19	90
Prospective	2	10
Data collection		
Single method	15	71
Semi‐structured interviews	9	43
Surveys	2	10
Clinical records	2	10
Video‐recordings of consultations	1	5
Ethnographic survey	1	5
Multi‐method	6	29
Semi‐structured interviews and focus groups	2	10
Semi‐structured interviews and surveys	2	10
Semi‐structured interviews, focus groups and surveys	2	10
Data analysis		
Single method	16	76
Thematic analysis	6	29
Grounded theory	5	24
Content analysis	3	14
Interpretative description	1	5
Statistical analysis	1	5
Multi‐method	5	24
Content analysis and statistical analysis	3	14
Thematic analysis and statistical analysis	2	10

The participants recruited included either GHP and other professionals (*n =* 8; 38%), patients (*n =* 6; 29%), or relatives present during GC (*n =* 1; 5%), with six studies (29%) including some combination of the previous groups. The level of detail provided regarding participant demographics varied across studies. In most studies that reported this information patient and relative participants were mostly women (*n =* 10; 48%), White (*n =* 7; 33%) and college‐educated or with partial college education (*n =* 5; 24%). Participants ages varied, with mean ages between 39 and 55 across studies. Among GHP, most were women (*n =* 7; 33%) and genetic counselors (*n =* 6; 29%) with a range of clinical experience from early‐career to over 20 years. Sample sizes varied from eight to 656 participants, with at least 2158 individuals (*n =* 1283 patients and relatives and *n =* 975 GHP and other professionals) and at least 1055 GC consultations represented across the 21 studies. One study (5%) did not state how many participants were involved (patients and GHP) and only five (24%) specified the number of consultations analyzed.

Table 3 presents the main findings of each study relating to the dimensions of the GC process and GHP's practices when addressing family communication.

**TABLE 3:** Dimensions of GC and practices to address family communication.

Dimensions of GC	References	Practices to address family communication			
		Guidance to inform the family	Materials to support communication	Psychosocial assessment	Additional support
Patient's experiences and perspectives	Brown et al. (2021)	Provide correct terminology to talk about the condition with relatives	Patient letters are sometimes used to facilitate family communication		Letters did not include all the resources discussed during appointments (e.g. contacts of GHP and other professionals)
	Cook et al. (2022)	Parents reported receiving some degree of support regarding communicating with their children about the condition, but none around communicating with them about the risk of psychiatric manifestations			
	Crook et al. (2022)	Inform patients of the condition's familial implications Give guidance surrounding plans for dissemination of information within the family	Folder with information, hotlines, websites and family letters	Assess family dynamics and barriers to communication	Offer further counseling and provide contacts of other healthcare professionals Reassure patients to facilitate adjustment to the information and family communication
	Gaff et al. (2005)	Inform patients of the implications of genetic testing for the individual and family Clarify which relatives are eligible for testing and how to inform them	Information booklets about the condition Personalized patient letters General family letters	Family dynamics were assessed by discussing the possible reactions of relatives	Encourage patients to discuss testing with family Occasional offers of follow‐up appointments to further discuss family communication
	Hudson et al. (2019)		Fact sheets and pamphlets with information and diagrams about the condition Family letters		A minority of participants sought and received support for family communication from genetic counselors or physicians before disclosure
	Kam et al. (2018)	Participants recalled GHP informing them about the inherited familial risks and the importance of conveying the information to their relatives			
	Lafrenière et al. (2013)	Highlighting the importance of sharing genetic information with at‐risk relatives Mention opportunities for communication (family gatherings)			Multidisciplinary support (GHP, oncologists, and psychiatrists), including encouragement to share information within the family
	Mendes and Sousa (2012)	Identify relatives to whom to disclose information Mention possible opportunities for family communication	Information pamphlets (used by participants to share information with estranged relatives)		Encourage information seeking (family health history) and sharing (genetic risk)
	Pedrazzani et al. (2022)	Highlight the importance of family communication, although not always clarifying which relatives need to be informed	Family letters		Offers of additional appointments Multidisciplinary support without coordination between providers Supportive attitude from GHP
GHP's roles and scope of practice	Clarke et al. (2005)	Reinforce the importance of communication with at‐risk relatives	Patient letters General family letters Written reminders to prompt disclosure	Explore reasons for nondisclosure and family dynamics	Involve multiple experienced GHP in discussions of family communication

	D'Audiffret Van Haecke and de Montgolfier (2016)	Extensive explanations about the condition and familial risk, while dispelling misconceptions Mention which relatives are at risk through the pedigree, who to inform, and the next steps to do so	Pro forma family letters	Assess family dynamics and patient preferences to adapt GC to patients and their families	Multidisciplinary support and referrals to psychologists Offer follow‐up appointments to discuss family communication Use relational circumvention
	Derbez et al. (2017)	Highlight the importance of family communication about genetic risk Identify at‐risk relatives using the pedigree Give examples of when and what information to share	Cover letter before GC (explaining that genetic testing may be offered to relatives) Lab reports with test results Patient letter (summary of test results and its implications, how to access GC, and monitoring/ prevention) Family letters (in case of nondisclosure)	Explore family dynamics, disclosure preferences, and reasons for nondisclosure	A multidisciplinary care team encourages information‐seeking and sharing in the family Referrals to psycho‐oncology for follow‐up support and contacts for relatives to access GC
	Fisher et al. (2017)	Highlight the importance of family communication by exploring family implications of test results Dispelling misconceptions about genetic risk Identifying at‐risk relatives, and tailoring disclosure to them	Patient letters (information summary about the condition, risk for specific relatives and recommendations for risk management and testing)	Explore family dynamics Explore patient preferences for disclosure and their reasons for nondisclosure	Relational circumvention Encouragement of information‐seeking and sharing, by reframing disclosure as “a pursuit of family survival” and validating previous instances of disclosure Involving relatives in GC with extra appointments
Forbes Shepherd et al. (2017)	Provide information on testing, genetic risk, and familial implications Identify at‐risk relatives using the pedigree Highlight the importance of family communication		Using an adaptable relational approach (covert, overt, or authoritative) Assess family dynamics, patient preferences, and reasons for nondisclosure Using hypothetical scenarios to promote reflection on the familial impact of nondisclosure	Encourage information seeking and sharing in the family, by reframing family disclosure as both part of genetic testing and as positive and empowering Relational circumvention

	Forrest et al. (2010)	Provide information about the genetic condition and its inheritance pattern Highlight the importance of family disclosure Identify at‐risk relatives	Patient letters Family letters (provided less frequently)		Encourage information‐seeking and sharing in the family Give patients their contacts and refer them to other professionals Offer follow‐up appointments for patients and relatives (in person and via telehealth)
	Gallo et al. (2010)	Discuss genetic risk, inheritance pattern, testing, implications for relatives and address misconceptions about these themes Highlight the importance of family disclosure Give guidance surrounding family disclosure with children Use educational techniques to guarantee understanding (asking if they have questions, asking focused questions, and reviewing information)	Sketches of genetic inheritance Booklets to facilitate information sharing with children Reliable information websites	Assessing family dynamics Explore parents', needs, emotional state, health literacy, and nonverbal communication during GC	Multidisciplinary support during GC Referrals to social workers for communication and family support in general
	Gorrie et al. (2018)	Provide information about the condition and its inheritance pattern, family implications, reproductive options, and cascade testing	Patient letters Family letters (explaining how to access carrier testing)		GC was sometimes articulated between the referring physician and a genetic counselor
	Makhnoon et al. (2021)	Information provision mostly mentioned cascade testing, followed by general descriptions of at‐risk relatives, and identification of specific relatives at risk The implications of genetic test results to the family and the importance of sharing this information were less frequently mentioned	Family letters Copy of genetic test results	Assess family dynamics, patient preferences for communication, and reasons for nondisclosure Adapting the relational approach to the patient (predominantly covert)	Logistical support for relatives including providing contacts of local resources to facilitate access to GC and testing
	Stol et al. (2010)	Medical geneticists advise patients to inform at‐risk relatives			
Young, Butow, Tucker, et al. (2019)		Family letters	Assess family communication dynamics Explore patient preferences and reasons for nondisclosure to ensure autonomy	Multidisciplinary support and referrals to other GHP and psychologists Explore family communication during follow‐up appointments Use relational circumvention

Young et al. (2020)	Educate and correct misconceptions on the terminology to discuss genetic risk and the familial impact of genetic results Highlight the importance of family communication Assess if patients retained information Give guidance on plans for family communication including when’, who’, ‘and how’ to disclose (including adapting the disclosure strategy for specific relatives)	Family letters with de‐identified personal information Booklets, pamphlets, and fact sheets (with information to disclose, techniques to facilitate disclosure, and testimonials of other families' experiences with risk disclosure) Websites with trustworthy information and social media to facilitate communication with relatives	Assess family communication dynamics Assess ability and reasons for disclosure and nondisclosure Use hypothetical scenarios and roleplay to explore the benefit/cost of nondisclosure to relatives and draw on previous experiences of disclosure	Provide follow‐up support (letters, in‐person appointments, or telehealth) Encourage information seeking and sharing, by normalizing family communication (with real‐life examples), advocating that relatives contact GC services, and reframing disclosure as beneficial for risk reduction Use relational circumvention

### 3.2 Dimensions of the GC process

All included studies analyzed how family communication of genetic information was addressed in GC by focusing on (i) patients' experiences and perspectives or (ii) GHP's roles and scope of practice surrounding family communication.

#### 3.2.1 Patient's experiences and perspectives

Nine studies (43%) collected patients' experiences with and perspectives on GHP's practices for addressing family communication during GC. In six (29%) studies, patients explicitly described how specific practices facilitated family communication. These included GHP clearly recommending sharing genetic information with at‐risk relatives (Kam et al., 2018; Lafrenière et al., 2013; Mendes & Sousa, 2012; Pedrazzani et al., 2022), providing materials with relevant information and resources (Crook et al., 2022; Gaff et al., 2005; Mendes & Sousa, 2012; Pedrazzani et al., 2022), as well as providing additional guidance or support at follow‐up (Pedrazzani et al., 2022) and when family communication challenges were present (Crook et al., 2022; Gaff et al., 2005; Kam et al., 2018; Lafrenière et al., 2013; Mendes & Sousa, 2012; Pedrazzani et al., 2022). Examples of discouraging practices were reported in four studies (19%). Some patients perceived that family communication was addressed too superficially or briefly (Cook et al., 2022; Crook et al., 2022; Lafrenière et al., 2013; Pedrazzani et al., 2022). Others reported that GHP did not always clarify which relatives would benefit from being informed (Pedrazzani et al., 2022), or consistently provided tools, materials, or documents to facilitate information sharing (Crook et al., 2022; Lafrenière et al., 2013). Additionally, sometimes multidisciplinary care teams lacked coordination when providing support (Pedrazzani et al., 2022).

In eight studies (38%), patients suggested practices to facilitate family communication in GC (Table S5). These described practical, family‐centered, and personalized support, including providing relevant information to be shared with relatives, communication resources, and follow‐up (Brown et al., 2021; Cook et al., 2022; Crook et al., 2022; Gaff et al., 2005; Hudson et al., 2019; Lafrenière et al., 2013; Pedrazzani et al., 2022). In four studies (19%), some patients favored a more proactive and directive approach during these discussions (Cook et al., 2022; Lafrenière et al., 2013; Mendes & Sousa, 2012; Pedrazzani et al., 2022), with two studies (10%) suggesting direct involvement of GHP in informing at‐risk relatives (Mendes & Sousa, 2012; Pedrazzani et al., 2022).

#### 3.2.2 GHP's roles and scope of practice

Twelve studies (57%) described the GHP's roles and scope of practice in addressing family communication of genetic information. The studies described the strategies, actions, and tools GHP used when discussing family communication, when they were used, and how (Clarke et al., 2005; D'Audiffret Van Haecke & de Montgolfier, 2016; Derbez et al., 2017; Fisher et al., 2017; Forbes Shepherd et al., 2017; Forrest et al., 2010; Gallo et al., 2010; Gorrie et al., 2018; Makhnoon et al., 2021; Young et al., 2020; Young, Butow, Tucker, et al., 2019). Two studies (10%) did this by observing GC appointments and analyzing the style and content of GHP–patient interactions (Derbez et al., 2017; Fisher et al., 2017).

Six studies (29%) reported that GHP preferred supporting patient‐mediated family communication instead of directly contacting relatives (Clarke et al., 2005; D'Audiffret Van Haecke & de Montgolfier, 2016; Derbez et al., 2017; Forrest et al., 2010; Stol et al., 2010; Young et al., 2020). In four studies (19%), GHP noted a need for more resources to address and support patients in family communication (Table S6) (Forrest et al., 2010; Gorrie et al., 2018; Young et al., 2020; Young, Butow, Tucker, et al., 2019).

### 3.3 How GHP addresses family communication

All studies mentioned that GHP discussed family communication of genetic information during GC. The descriptions of how that was addressed in practice varied, with two studies (10%) mentioning that the topic was addressed without specifying the practices involved (Cook et al., 2022; Stol et al., 2010). When studies detailed GHP's practices, these included providing (i) guidance to inform the family (ii) materials to support communication, (iii) psychosocial assessment, and (iv) additional support.^1^ Eighteen studies (86%) described that GHP adopted more than one of these practices (Brown et al., 2021; Clarke et al., 2005; Crook et al., 2022; D'Audiffret Van Haecke & de Montgolfier, 2016; Derbez et al., 2017; Fisher et al., 2017; Forbes Shepherd et al., 2017; Forrest et al., 2010; Gaff et al., 2005; Gallo et al., 2010; Gorrie et al., 2018; Hudson et al., 2019; Lafrenière et al., 2013; Makhnoon et al., 2021; Mendes & Sousa, 2012; Pedrazzani et al., 2022; Young et al., 2020; Young, Butow, Tucker, et al., 2019), while three studies (14%) described that GHP only provided guidance to inform the family (Cook et al., 2022; Kam et al., 2018; Stol et al., 2010).

Sixteen studies (76%) reported contextual factors influencing how GHP address family communication (Table S7). Some encouraged these discussions during GC, namely disease and genetic variant characteristics (*n =* 3; 14%; Forrest et al., 2010; Gallo et al., 2010; Young et al., 2020), in‐person appointments happening in a private, quiet, and suitable space (*n =* 2; 10%) (Gallo et al., 2010; Young, Butow, Tucker, et al., 2019), consent forms that addressed sharing results with at‐risk relatives (*n =* 3; 14%; D'Audiffret Van Haecke & de Montgolfier, 2016; Derbez et al., 2017; Forbes Shepherd et al., 2017), providing multiple appointments (*n =* 4; 19%; Clarke et al., 2005; Gorrie et al., 2018; Makhnoon et al., 2021; Young, Butow, Tucker, et al., 2019) and materials in different formats to support communication (*n =* 1; 5%; Brown et al., 2021). Similarly, discussions about family communication between GHP and patients were also promoted by GHP preferring patient‐mediated communication with relatives (*n =* 6; 29%; Crook et al., 2022; D'Audiffret Van Haecke & de Montgolfier, 2016; Lafrenière et al., 2013; Makhnoon et al., 2021; Stol et al., 2010; Young et al., 2020), offering patients support and perceiving family communication discussions as an integral part of GC (*n =* 2; 10%; Forrest et al., 2010; Gallo et al., 2010).

When family communication was addressed in GC varied, with some GHPs preferring to discuss it after disclosing test results (*n =* 2; 10%; D'Audiffret Van Haecke & de Montgolfier, 2016; Gorrie et al., 2018), while others discussed it since the first contact (*n =* 6; 29%) (Clarke et al., 2005; D'Audiffret Van Haecke & de Montgolfier, 2016; Derbez et al., 2017; Gaff et al., 2005; Young et al., 2020; Young, Butow, Tucker, et al., 2019). The emphasis given by GHPs to family communication also varied, with descriptions of both superficial (*n =* 4; 19%; Cook et al., 2022; Crook et al., 2022; Lafrenière et al., 2013; Pedrazzani et al., 2022) and in‐depth discussions (*n =* 6; 29%) (D'Audiffret Van Haecke & de Montgolfier, 2016; Derbez et al., 2017; Forbes Shepherd et al., 2017; Pedrazzani et al., 2022; Young et al., 2020; Young, Butow, Tucker, et al., 2019).

#### 3.3.1 Guidance to inform the family

Nineteen studies (90%) described the guidance that GHP provided to patients for facilitating their communication of accurate information to at‐risk relatives, including (i) educating and addressing patient's misconceptions and its implications on relatives' genetic risk, (ii) highlighting the importance of family communication, (iii) identifying at‐risk relatives, and (iv) addressing dissemination plans.

Thirteen studies (62%) reported that GHP educated patients on the genetic condition's impact for the family. This could include explaining the inheritance pattern and the implications to relatives' genetic risk (*n =* 8; 38%) (Derbez et al., 2017; Forbes Shepherd et al., 2017; Forrest et al., 2010; Gaff et al., 2005; Gallo et al., 2010; Gorrie et al., 2018; Kam et al., 2018; Makhnoon et al., 2021), dispelling patients' misconceptions (*n =* 4; 19%) (D'Audiffret Van Haecke & de Montgolfier, 2016; Fisher et al., 2017; Gallo et al., 2010; Young et al., 2020) and teaching the correct terminology to facilitate family communication (*n =* 3; 14%) (Brown et al., 2021; Gallo et al., 2010; Young et al., 2020). Five studies (24%) clarified that information about relatives' genetic risk was provided alongside explanations of symptoms, surveillance, therapeutic options, and genetic testing for the patient and their at‐risk relatives (Crook et al., 2022; Gaff et al., 2005; Gallo et al., 2010; Gorrie et al., 2018; Makhnoon et al., 2021). To ensure patient understanding, GHP explored patient questions, asked questions focused on important information shared during GC, and repeatedly reviewed information over time (*n =* 3; 14%) (D'Audiffret Van Haecke & de Montgolfier, 2016; Gallo et al., 2010; Young et al., 2020).

In 14 studies (67%), GHP highlighted the importance of informing relatives about their genetic risk by ensuring the patient was aware that genetic test results have implications for relatives (Clarke et al., 2005; Crook et al., 2022; Derbez et al., 2017; Fisher et al., 2017; Forbes Shepherd et al., 2017; Forrest et al., 2010; Gaff et al., 2005; Gallo et al., 2010; Kam et al., 2018; Lafrenière et al., 2013; Makhnoon et al., 2021; Pedrazzani et al., 2022; Stol et al., 2010; Young et al., 2020). One study reported this was done by the overwhelming majority of GHP (Forrest et al., 2010). However, in three of the studies (14%) that reported this practice, not all GHP explained or emphasized why informing relatives was important (Lafrenière et al., 2013; Makhnoon et al., 2021; Pedrazzani et al., 2022) or identified which relatives to inform (Pedrazzani et al., 2022). One study reported that while an overwhelming majority of GHP consultation notes mentioned discussing cascade genetic testing with patients, few noted advising patients to share lab reports with at‐risk relatives so they could access GC (Makhnoon et al., 2021).

Nine studies (43%) reported that GHP identified at‐risk relatives (D'Audiffret Van Haecke & de Montgolfier, 2016; Derbez et al., 2017; Fisher et al., 2017; Forbes Shepherd et al., 2017; Forrest et al., 2010; Gaff et al., 2005; Makhnoon et al., 2021; Mendes & Sousa, 2012; Young et al., 2020). One study (5%) specified this was done either through generic descriptions of relatives at risk (e.g. first‐degree relatives) and less frequently specifying the type of kinship that could be at risk (e.g. siblings) (Makhnoon et al., 2021). Three studies (14%) reported using the pedigree as a visual aid to help identify relatives at risk (D'Audiffret Van Haecke & de Montgolfier, 2016; Derbez et al., 2017; Forbes Shepherd et al., 2017).

Nine studies (43%) described that GHP addressed family communication dissemination plans, including the appropriate timing (*n =* 3; 14%) (Derbez et al., 2017; Mendes & Sousa, 2012; Young et al., 2020), setting (*n =* 1; 5%) (Lafrenière et al., 2013), content (*n =* 3; 14%) (Crook et al., 2022; Gaff et al., 2005; Gallo et al., 2010), and upcoming tasks in the disclosure process (*n =* 1; 5%; D'Audiffret Van Haecke & de Montgolfier, 2016). Three studies (14%) (Fisher et al., 2017; Gallo et al., 2010; Young et al., 2020) reported that GHP advised patients on how to convey the information to a particular relative.

#### 3.3.2 Materials to support communication

Sixteen studies (76%) described the written materials provided to patients by GHP. These were either specifically designed to aid in family communication or not intended for that purpose but still used by patients to facilitate communication. Those included (i) educational materials and information packs, (ii) family letters, (iii) other letters and documents, and (iv) online resources.

Educational materials and information packs included pamphlets, booklets, and fact sheets with information summaries and diagrams. Six studies (29%) reported using them, and their content included information about the condition, inheritance pattern, genetic risk for relatives, and genetic testing (Crook et al., 2022; Gaff et al., 2005; Gallo et al., 2010; Hudson et al., 2019; Mendes & Sousa, 2012; Young et al., 2020). One study also identified a glossary, techniques to facilitate communication, and other families' experiences with information sharing (*n =* 1; 5%; Young et al., 2020).

In 12 studies (57%), patients were given family letters during GC to share with at‐risk relatives. They included information on the condition, genetic risk for relatives, and how to access GC and testing (Clarke et al., 2005; Crook et al., 2022; D'Audiffret Van Haecke & de Montgolfier, 2016; Derbez et al., 2017; Forrest et al., 2010; Gaff et al., 2005; Gorrie et al., 2018; Hudson et al., 2019; Makhnoon et al., 2021; Pedrazzani et al., 2022; Young et al., 2020; Young, Butow, Tucker, et al., 2019). Three studies (14%) described them as general letters that could be given to any relative (Clarke et al., 2005; D'Audiffret Van Haecke & de Montgolfier, 2016; Gaff et al., 2005), while one study (5%) described them as personalized to specific relatives (Makhnoon et al., 2021). Three studies (14%) clarified that the identity of tested relatives was usually not disclosed in the letters (Clarke et al., 2005; Gaff et al., 2005; Young et al., 2020). Three studies (10%) clarified that when direct communication from GHP to relatives was an option, it could be done through letters (Derbez et al., 2017; Forrest et al., 2010).

Six studies (29%) described using patient letters to help guide communication with the family (Brown et al., 2021; Clarke et al., 2005; Derbez et al., 2017; Fisher et al., 2017; Forrest et al., 2010; Gaff et al., 2005). Unlike family letters, these were intended only for patients and summarized the information provided during GC. In one study (5%), cover letters were provided to patients before GC to clarify that relatives could also be offered genetic testing if necessary (Derbez et al., 2017). Other studies (5%) reported that written prompts were sent at follow‐up to remind patients of the importance of family communication (Clarke et al., 2005). Two studies (10%) specified that lab reports with genetic test results were also shared with patients, as copies would be needed for relatives to access genetic testing (Derbez et al., 2017; Makhnoon et al., 2021).

Three studies (14%) reported that online resources such as social media and GHP‐approved websites were used to facilitate information sharing with distant or estranged relatives (Crook et al., 2022; Gallo et al., 2010; Young et al., 2020).

#### 3.3.3 Psychosocial assessment

Eleven studies (52%) described GHP exploring how psychosocial factors could influence a patient's ability to understand and cope with the need for family communication. This included (i) understanding patients' preferences for communication, (ii) exploring family dynamics, (iii) discussing reasons for noncommunication, (iv) employing hypothetical scenarios, and (v) adapting one's relational approach to each patient.

Eight studies (38%) reported that GHP sought to understand how patients felt about communicating with relatives. This included exploring patients' values, needs, motivations, autonomy, perceptions of responsibility toward relatives, and ability to cope with family communication (D'Audiffret Van Haecke & de Montgolfier, 2016; Derbez et al., 2017; Fisher et al., 2017; Forbes Shepherd et al., 2017; Gallo et al., 2010; Makhnoon et al., 2021; Young et al., 2020; Young, Butow, Tucker, et al., 2019), with one study mentioning GHP being attentive to nonverbal cues (Gallo et al., 2010).

Eleven studies (52%) described GHP assessing family dynamics to tailor their approach and patient support (Clarke et al., 2005; Crook et al., 2022; D'Audiffret Van Haecke & de Montgolfier, 2016; Derbez et al., 2017; Fisher et al., 2017; Forbes Shepherd et al., 2017; Gaff et al., 2005; Gallo et al., 2010; Makhnoon et al., 2021; Young et al., 2020; Young, Butow, Tucker, et al., 2019). One study (5%) clarified this involved exploring positive and negative relational patterns with relatives (Makhnoon et al., 2021), while another study (5%) described exploring support dynamics (Gallo et al., 2010). Timing varied, with one study (5%) noting family dynamics sometimes were explored during pedigree construction (Forbes Shepherd et al., 2017).

Eight studies (38%) reported GHP exploring patients' motivations in cases of possible nondisclosure within the family (Clarke et al., 2005; Crook et al., 2022; Derbez et al., 2017; Fisher et al., 2017; Forbes Shepherd et al., 2017; Makhnoon et al., 2021; Young et al., 2020; Young, Butow, Tucker, et al., 2019). Three studies (14%) mentioned GHP using hypothetical scenarios to promote relational empathy and reflection about the potential reactions of relatives to disclosure (*n =* 1; 5%) (Gaff et al., 2005) and the implications of nondisclosure (*n =* 2; 10%) (Forbes Shepherd et al., 2017; Young et al., 2020).

Two studies (10%) reported that GHP adapted their relational approach to address family communication, changing their level of directiveness to suit each patient (Forbes Shepherd et al., 2017; Makhnoon et al., 2021).

#### 3.3.4 Additional support

Eighteen studies (86%) described forms of additional support provided by GHP to facilitate family communication, including (i) offering extra appointments, (ii) liaising with healthcare professionals from other specialties, (iii) encouraging information seeking and sharing, and (iv) relational circumvention.

Ten studies (47%) described GHP offering extra appointments (Crook et al., 2022; D'Audiffret Van Haecke & de Montgolfier, 2016; Derbez et al., 2017; Fisher et al., 2017; Forrest et al., 2010; Gaff et al., 2005; Hudson et al., 2019; Pedrazzani et al., 2022; Young et al., 2020; Young, Butow, Tucker, et al., 2019), although three studies (14%) reported that this was only done if patients requested it (Crook et al., 2022; Hudson et al., 2019; Pedrazzani et al., 2022). Six studies (29%) specified extra appointments were usually follow‐up (D'Audiffret Van Haecke & de Montgolfier, 2016; Derbez et al., 2017; Forrest et al., 2010; Gaff et al., 2005; Young et al., 2020; Young, Butow, Tucker, et al., 2019), while three studies (14%) described them as either individual or family (in‐person or telehealth) appointments (Fisher et al., 2017; Forrest et al., 2010; Young et al., 2020). Topics discussed varied: four studies (19%) described them as covering mostly family communication (Crook et al., 2022; D'Audiffret Van Haecke & de Montgolfier, 2016; Pedrazzani et al., 2022; Young, Butow, Tucker, et al., 2019), while one (5%) described them as discussing various topics (Derbez et al., 2017).

Multidisciplinary support was mentioned in 12 studies (57%) and involved collaboration between multiple professionals to help patients with family communication (Brown et al., 2021; Clarke et al., 2005; Crook et al., 2022; D'Audiffret Van Haecke & de Montgolfier, 2016; Derbez et al., 2017; Forrest et al., 2010; Gallo et al., 2010; Gorrie et al., 2018; Lafrenière et al., 2013; Makhnoon et al., 2021; Pedrazzani et al., 2022; Young, Butow, Tucker, et al., 2019). One study (5%) described constraints in coordinating this support (Pedrazzani et al., 2022). Referrals to other professionals for support involved GHP scheduling appointments (*n =* 2; 10%) (Gallo et al., 2010; Young, Butow, Tucker, et al., 2019), providing contacts of specialists and information about local resources (*n =* 3; 14%) (Brown et al., 2021; Crook et al., 2022; Makhnoon et al., 2021), or both (*n =* 2; 10%) Derbez et al., 2017; Forrest et al., 2010).

Ten studies (47%) described that GHP encouraged information sharing and seeking within the family (Crook et al., 2022; Derbez et al., 2017; Fisher et al., 2017; Forbes Shepherd et al., 2017; Forrest et al., 2010; Gaff et al., 2005; Lafrenière et al., 2013; Mendes & Sousa, 2012; Pedrazzani et al., 2022; Young et al., 2020). Five studies (24%) clarified strategies employed by GHP to normalize family communication, including framing it as necessary and positive (*n =* 2; 10%) (Fisher et al., 2017; Forbes Shepherd et al., 2017), reassuring patients (*n =* 2; 10%) (Fisher et al., 2017; Forbes Shepherd et al., 2017), and adopting a supportive attitude (*n =* 3; 14%) (Crook et al., 2022; Pedrazzani et al., 2022; Young et al., 2020).

Five studies (24%) mentioned GHP using relational circumvention to support family communication (Forbes Shepherd et al., 2017), identifying persons (relatives, healthcare professionals, important members of the community, among others) that could facilitate communication with difficult‐to‐reach relatives (D'Audiffret Van Haecke & de Montgolfier, 2016; Fisher et al., 2017; Forbes Shepherd et al., 2017; Young et al., 2020; Young, Butow, Tucker, et al., 2019).

## 4 DISCUSSION

This scoping review aimed to map the methodological approaches, key dimensions of the GC process, and research evidence from process studies that examined how family communication of genetic information is addressed in GC.

Process studies allow for a nuanced understanding of which strategies and skills GHP use, when and how they are implemented in clinical settings, and how they are experienced (Biesecker & Peters, 2001). They also help clarify decision‐making processes, relational dynamics, and contextual factors that shape how GHP support patients (Biesecker & Peters, 2001; Paul et al., 2015). Synthesizing this evidence is crucial as no previous reviews have focused exclusively on process studies that explore how family communication is addressed in GC. In doing so, this review adds to the literature with insights that go beyond descriptions of ideal professional practices found in guidelines and normative documents (Forrest et al., 2007; Phillips et al., 2021), retrospective accounts by GHP in intervention studies, assessments of effectiveness (Ballard et al., 2023; Baroutsou et al., 2021; Law et al., 2022; Young et al., 2023; Zhao et al., 2022), and evaluations of GC outcomes (Cirino et al., 2022; Katz et al., 2024).

Our review complements a previous systematic review (Mendes et al., 2016) by including studies published from 2014 onwards, offering updated insights into current practices used by GHP to support patients and capturing key developments in genomic medicine since then. Additionally, by including the perspectives of GHP, patients, and relatives, our review provides a holistic view of the care delivered in GC, highlighting the processes, strategies, and challenges involved in addressing family communication of genetic information in practice.

The main findings show that most reviewed studies included hereditary cancer syndromes, used qualitative and retrospective designs, and explored the perspectives of GHP and patients, with few studies including the perspectives of patients' relatives, which highlights a possible research gap. Most studies were conducted by multidisciplinary research teams and published in the last 10 years, suggesting a growing interest in this topic, particularly regarding oncogenetics (Bowen et al., 2021; Fox et al., 2021; Wallgren et al., 2021; Zakaria et al., 2023), which is the most prevalent setting in GC process studies (Paneque et al., 2012).

The reviewed studies suggest that GHP commonly address the importance of communicating genetic information to relatives. This aligns with GC practice literature (Clarke et al., 1996; Meiser et al., 2008; Mendes et al., 2016; Paneque et al., 2012; Paul et al., 2015) and interventions to facilitate family communication (Ballard et al., 2023; Baroutsou et al., 2021; Zhao et al., 2022). Nevertheless, reviews clarifying how family communication is addressed by GHP are scarce and provide only limited descriptions of GC practice (Mendes et al., 2016). Providing patients with guidance to inform the family was the most frequently adopted practice by GHP. This included educating patients on the importance of family communication, identifying at‐risk relatives, and discussing dissemination plans (Brown et al., 2021; Clarke et al., 2005; Cook et al., 2022; Crook et al., 2022; D'Audiffret Van Haecke & de Montgolfier, 2016; Derbez et al., 2017; Fisher et al., 2017; Forbes Shepherd et al., 2017; Forrest et al., 2010; Gaff et al., 2005; Gallo et al., 2010; Gorrie et al., 2018; Kam et al., 2018; Lafrenière et al., 2013; Makhnoon et al., 2021; Mendes & Sousa, 2012; Pedrazzani et al., 2022; Stol et al., 2010; Young et al., 2020). Written materials to support family communication, particularly family letters and educational resources, also often aimed to promote effective information sharing (Brown et al., 2021; Clarke et al., 2005; Crook et al., 2022; D'Audiffret Van Haecke & de Montgolfier, 2016; Derbez et al., 2017; Fisher et al., 2017; Forrest et al., 2010; Gaff et al., 2005; Gallo et al., 2010; Gorrie et al., 2018; Hudson et al., 2019; Makhnoon et al., 2021; Mendes & Sousa, 2012; Pedrazzani et al., 2022; Young et al., 2020; Young, Butow, Tucker, et al., 2019). This focus on information provision aligns with the teaching model of GC (Kessler, 1997), which is observed in most studies exploring GC practice (Meiser et al., 2008; Paul et al., 2015). The teaching model emphasizes the effective communication of information to the patient, including the implications for relatives and the relevance of informing them of their risk (Clarke et al., 1996; Kessler, 1997; Paul et al., 2015). In this model, GHP typically hold more authority in the relationship, with conversations predominantly led by them and minimal input from the patient (Biesecker, 2020; Clarke et al., 1996; Kessler, 1997; Meiser et al., 2008; Paul et al., 2015).

The studies also report that GHP less commonly assessed patients' ability to share information with at‐risk relatives. This included exploring family dynamics, understanding patient preferences for family communication, and using hypothetical scenarios to promote patient insight, identify resources, and personalize GC (Clarke et al., 2005; Crook et al., 2022; D'Audiffret Van Haecke & de Montgolfier, 2016; Derbez et al., 2017; Fisher et al., 2017; Forbes Shepherd et al., 2017; Gaff et al., 2005; Gallo et al., 2010; Makhnoon et al., 2021; Young et al., 2020; Young, Butow, Tucker, et al., 2019). Several studies reported on patient decision‐making about informing the family (Clarke et al., 2005; Crook et al., 2022; Derbez et al., 2017; Fisher et al., 2017; Forbes Shepherd et al., 2017; Hudson et al., 2019; Lafrenière et al., 2013; Pedrazzani et al., 2022; Young et al., 2020; Young, Butow, Tucker, et al., 2019); however, GHP primarily explored this when patients shared they do not want to inform relatives (Clarke et al., 2005; Derbez et al., 2017; Fisher et al., 2017; Forbes Shepherd et al., 2017; Young et al., 2020; Young, Butow, Tucker, et al., 2019). This suggests that, in the reviewed studies, psychosocial strategies aimed at facilitating family disclosure, tailoring support to the patient, and enhancing their autonomy by considering their preferences and family dynamics, are significantly less prevalent than support based solely on information provision (Brown et al., 2021; Clarke et al., 2005; Cook et al., 2022; Crook et al., 2022; D'Audiffret Van Haecke & de Montgolfier, 2016; Derbez et al., 2017; Fisher et al., 2017; Forbes Shepherd et al., 2017; Forrest et al., 2010; Gaff et al., 2005; Gallo et al., 2010; Gorrie et al., 2018; Kam et al., 2018; Lafrenière et al., 2013; Makhnoon et al., 2021; Mendes & Sousa, 2012; Pedrazzani et al., 2022; Stol et al., 2010; Young et al., 2020). This imbalance raises questions about whether the practices described in process studies align with professional guidelines (Phillips et al., 2021) and consensus reports (Phillips et al., 2025), which recommend greater support during GC. Although information provision and education are essential to help patients prepare for family communication, they often also require psychosocial support to navigate emotional and relational challenges it involves (Afaya et al., 2024; Elrick et al., 2017; Marleen van den Heuvel et al., 2020; Young, Butow, Tucker, et al., 2019). GHP can assist by offering nuanced, emotionally and relationally sensitive guidance (Lumpkins et al., 2023; Mendes et al., 2018; Phillips et al., 2025).

Most studies in our review reported that GHP provided patients with additional support to facilitate family communication, including multidisciplinary support, encouraging information seeking and sharing with relatives, relational circumvention and extra appointments (Brown et al., 2021; Clarke et al., 2005; Crook et al., 2022; D'Audiffret Van Haecke & de Montgolfier, 2016; Derbez et al., 2017; Fisher et al., 2017; Forbes Shepherd et al., 2017; Forrest et al., 2010; Gaff et al., 2005; Gallo et al., 2010; Gorrie et al., 2018; Hudson et al., 2019; Lafrenière et al., 2013; Makhnoon et al., 2021; Mendes & Sousa, 2012; Pedrazzani et al., 2022; Young et al., 2020; Young, Butow, Tucker, et al., 2019). Additionally, GHP's perceptions that discussions about family communication are an integral part of GC, along with offering their support, were factors that promoted dialogue on the topic (Forrest et al., 2010). Employing both psychosocial assessment and additional support practices aligns with the counseling model of GC (Kessler, 1997). This model prioritizes patient‐centered care focusing on responding to patient needs, relieving distress, supporting self‐efficacy, and effective coping by identifying patient resources (Biesecker, 2020; Kessler, 1997). According to this model, patients are supported through collaborative work that focuses on solutions consistent with their needs and values (Hartmann et al., 2015).

Overall, our review suggests that the approaches to family communication in GC research draw on both teaching and counseling models, although with greater emphasis on the former. This is consistent with GC models that integrate both teaching and counseling principles, such as the psychotherapeutic model (Austin et al., 2014; Biesecker, 2020) and the reciprocal engagement model (REM) (Hartmann et al., 2015; Redlinger‐Grosse et al., 2017; Veach et al., 2007). The psychotherapeutic model integrates psychotherapeutic principles into GC to help individuals adapt to the implications of genetic conditions through short‐term focused support (Austin et al., 2014). Similarly, the REM aligns with patient‐centered care, as patient education and information provision depend on the GHP–patient relationship and are shaped by it (Veach et al., 2007).

Descriptions of practices to address family communication in the reviewed studies differed between GHP (Crook et al., 2022; D'Audiffret Van Haecke & de Montgolfier, 2016; Lafrenière et al., 2013; Makhnoon et al., 2021; Pedrazzani et al., 2022; Young et al., 2020). Several studies reported that clinical practice was dependent on contextual factors of service provision, such as its structure and setting, GHP's practice preferences, and the resources available (Brown et al., 2021; Clarke et al., 2005; Crook et al., 2022; D'Audiffret Van Haecke & de Montgolfier, 2016; Derbez et al., 2017; Forbes Shepherd et al., 2017; Forrest et al., 2010; Gaff et al., 2005; Gallo et al., 2010; Gorrie et al., 2018; Lafrenière et al., 2013; Makhnoon et al., 2021; Pedrazzani et al., 2022; Stol et al., 2010; Young et al., 2020; Young, Butow, Tucker, et al., 2019). While this heterogeneity may reflect discrepancies and a lack of clarity in current guidelines (Forrest et al., 2007; Phillips et al., 2021), it could also stem from an increased demand for GC that exceeds existing resources (Dusic et al., 2022; Ormond et al., 2024; Raspa et al., 2021). Some GHP reported lacking the resources necessary to provide follow‐up support (Forrest et al., 2010; Gorrie et al., 2018; Young et al., 2020).

Less than a third of the studies in this review reported when family communication was discussed with patients (Clarke et al., 2005; D'Audiffret Van Haecke & de Montgolfier, 2016; Derbez et al., 2017; Gaff et al., 2005; Gorrie et al., 2018; Young et al., 2020; Young, Butow, Tucker, et al., 2019) with most of those reporting GHP addressing it throughout GC (Clarke et al., 2005; D'Audiffret Van Haecke & de Montgolfier, 2016; Derbez et al., 2017; Gaff et al., 2005; Young et al., 2020; Young, Butow, Tucker, et al., 2019). This is especially helpful, as limited discussions may prevent patients from articulating their preferences and support needs, since they are often unaware of the challenges in family communication until they have to engage in it (Marleen van den Heuvel et al., 2020; Young, Butow, Rhodes, et al., 2019). Addressing these issues early and providing ongoing support is key to supporting effective family communication in GC (Marleen van den Heuvel et al., 2020; Young, Butow, Rhodes, et al., 2019). Some of the reviewed studies reflect this, as some patients either requested or suggested additional support to facilitate family communication (Cook et al., 2022; Crook et al., 2022; Gaff et al., 2005; Hudson et al., 2019; Lafrenière et al., 2013; Pedrazzani et al., 2022). This aligns with studies that describe resources preferred by patients to assist with family communication (Cragun et al., 2021), as well as their recommendations to enhance support. These include ongoing, forthright, and family‐centered discussions, provision of physical and web‐based resources, guidance on how to broach the topic, and the option of direct communication with relatives by the GHP (Marleen van den Heuvel et al., 2020; Phillips et al., 2025; Pollard et al., 2020).

A comparison between patient‐suggested strategies and reported GHP practices reveals both areas of alignment and divergence. Consistent with patient suggestions (Crook et al., 2022; Hudson et al., 2019; Lafrenière et al., 2013), most of the reviewed studies described GHP providing education and emphasizing the importance of family communication (Brown et al., 2021; Clarke et al., 2005; Cook et al., 2022; Crook et al., 2022; D'Audiffret Van Haecke & de Montgolfier, 2016; Derbez et al., 2017; Fisher et al., 2017; Forbes Shepherd et al., 2017; Forrest et al., 2010; Gaff et al., 2005; Gallo et al., 2010; Gorrie et al., 2018; Kam et al., 2018; Lafrenière et al., 2013; Makhnoon et al., 2021; Pedrazzani et al., 2022; Stol et al., 2010; Young et al., 2020). However, patients also expressed a desire for more practical and concrete support, such as communication planning and dissemination strategies (Cook et al., 2022; Gaff et al., 2005), which were addressed in fewer than half of the studies (Cook et al., 2022; D'Audiffret Van Haecke & de Montgolfier, 2016; Derbez et al., 2017; Fisher et al., 2017; Gaff et al., 2005; Gallo et al., 2010; Lafrenière et al., 2013; Mendes & Sousa, 2012; Young et al., 2020). Additionally, personalized guidance (Fisher et al., 2017; Gallo et al., 2010; Young et al., 2020) and information on access to local resources were rarely included (Brown et al., 2021; Crook et al., 2022; Derbez et al., 2017; Forrest et al., 2010; Makhnoon et al., 2021).

Although most studies reported GHP providing written materials to assist patients (Brown et al., 2021; Clarke et al., 2005; Crook et al., 2022; D'Audiffret Van Haecke & de Montgolfier, 2016; Derbez et al., 2017; Fisher et al., 2017; Forrest et al., 2010; Gaff et al., 2005; Gallo et al., 2010; Gorrie et al., 2018; Hudson et al., 2019; Makhnoon et al., 2021; Mendes & Sousa, 2012; Pedrazzani et al., 2022; Young et al., 2020; Young, Butow, Tucker, et al., 2019), few of these materials were tailored, such as personalized family (Makhnoon et al., 2021) or patient letters (Gaff et al., 2005). Lists of at‐risk relatives or educational videos roleplaying family communication were not included in the reviewed studies (Cook et al., 2022; Gaff et al., 2005). While some studies described identifying at‐risk relatives (D'Audiffret Van Haecke & de Montgolfier, 2016; Derbez et al., 2017; Fisher et al., 2017; Forbes Shepherd et al., 2017; Forrest et al., 2010; Gaff et al., 2005; Makhnoon et al., 2021; Mendes & Sousa, 2012; Young et al., 2020) and mentioned using roleplays or testimonials during appointments (Young et al., 2020), these were not provided in formats that patients could access afterward, such as online resources, which were underreported (Crook et al., 2022; Gallo et al., 2010; Young et al., 2020). Although educational roleplay videos have demonstrated effectiveness in providing information and promoting empowerment across health literacy levels (Hurtado‐de‐Mendoza et al., 2020, 2021), their use to support family communication was unexplored in our review. Combined with the limited reporting of follow‐up consultations (D'Audiffret Van Haecke & de Montgolfier, 2016; Derbez et al., 2017; Forrest et al., 2010; Gaff et al., 2005; Young et al., 2020; Young, Butow, Tucker, et al., 2019) and patient‐reported needs for ongoing support (Cook et al., 2022; Crook et al., 2022; Lafrenière et al., 2013; Pedrazzani et al., 2022), this highlights a significant research gap in developing scalable, accessible methods of postconsultation support.

Psychosocial assessments were described in over half of the studies, enabling some degree of tailoring in communication approaches. However, their implementation appeared inconsistent, sometimes occurring opportunistically rather than as a routine component of GC. The studies varied in terms of which specific psychosocial factors were assessed by GHP (e.g., coping skills, nonverbal cues) (D'Audiffret Van Haecke & de Montgolfier, 2016; Derbez et al., 2017; Fisher et al., 2017; Forbes Shepherd et al., 2017; Gallo et al., 2010; Makhnoon et al., 2021; Young et al., 2020; Young, Butow, Tucker, et al., 2019), the methods used (e.g., hypothetical scenarios (Forbes Shepherd et al., 2017; Gaff et al., 2005; Young et al., 2020), adaptive relational approach (Forbes Shepherd et al., 2017; Makhnoon et al., 2021)), and the timing of assessments (Forbes Shepherd et al., 2017).

Additionally, some patients suggested that GHP could be more forthright and directive when discussing family communication (Cook et al., 2022; Lafrenière et al., 2013; Pedrazzani et al., 2022), although such approaches were rarely reported in the included studies (Forbes Shepherd et al., 2017). Other patients also expressed a preference for more direct GHP involvement in informing relatives (Mendes & Sousa, 2012; Pedrazzani et al., 2022), aligning with findings from studies conducted with people living with inherited conditions and in the general population (Marleen van den Heuvel et al., 2020; Ribeiro et al., 2025). Although a few of the included studies reported direct contact between GHP and relatives as a viable option (D'Audiffret Van Haecke & de Montgolfier, 2016; Derbez et al., 2017; Forrest et al., 2010), most studies indicated that GHP had a preference for patient‐mediated communication (Clarke et al., 2005; D'Audiffret Van Haecke & de Montgolfier, 2016; Derbez et al., 2017; Forrest et al., 2010; Stol et al., 2010; Young et al., 2020). Notably, some studies reported the use of family GC appointments (Fisher et al., 2017; Forrest et al., 2010; Young et al., 2020), suggesting a potential area for further research to explore models that balance patient autonomy with greater GHP involvement in family communication.

### 4.1 Limitations

This review has several limitations. First, the reviewed studies may reflect publication bias, as studies that were not published or peer‐reviewed were excluded. Second, the search was limited to studies published in English, French, Portuguese, or Spanish; as a result, some relevant literature may have been missed despite our efforts to use a comprehensive range of search terms. Third, reviewed studies were primarily conducted in Australia, North America, and Europe, with most focusing GC in the context of oncogenetics.

Additionally, samples also disproportionately included White and female participants across studies. Most relatives and patients also had at least some college education, indicating potential selection biases. Consequently, the findings may not reflect clinical practice in other contexts or the perspectives and experiences of other demographic groups in our study population. Moreover, not all studies specified all the types of GHP involved in GC, which limits the ability to compare practices across different GHP. Finally, in accordance with the JBI recommendations for scoping reviews (Aromataris et al., 2024; Peters et al., 2021; Tricco et al., 2018), we have refrained from drawing implications for practice or policy, as we did not evaluate the quality of the included studies.

## 5 CONCLUSION

To the best of our knowledge, this is the first literature review to focus on GC practice regarding family communication of genetic information through the analysis of process studies. The studies included in this review primarily employed a qualitative, retrospective design and mainly focused on GC within the context of hereditary cancer.

The findings show a degree of heterogeneity in how this topic is approached in their practice. The reviewed studies demonstrate GHP consistently inform patients about their relatives' genetic risk, emphasize the importance of such communication, and provide written resources and additional support to facilitate family communication. However, this review also indicates that GHP assess patients' ability to communicate information with relatives less frequently.

Our findings offer insight into how family communication is addressed in GC, and its alignment within the scope of practice of GHP as well as the models of GC they adopt. These insights may help inform future research on the practical application of integrated GC models. In particular, the observed emphasis on the teaching model, alongside the relatively limited use of counseling techniques, raises important questions about how GHP manage the balance between providing information and offering psychosocial support. This highlights areas where further research could support the evolution of GC approaches regarding the family communication of genetic information, addressing growing demand, service constraints, and diverse patient needs.

The reported divergences between patient‐suggested strategies and reported GHP practices suggest a gap between patient expectations and current practice. This highlights the need for further research to explore how GC strategies can be adapted to better align with the communication needs, preferences, and circumstances of patients and their families. Additionally, future studies could prioritize prospective process studies to investigate how family communication of genetic information is addressed in GC using observational data, with a broader range of demographics and clinical contexts. This would help explore the influence of GC on patient decision‐making regarding family disclosure while also providing insight into patient‐GHP interactions during GC. These insights would be valuable for healthcare services, GHP, policymakers, and at‐risk families, as they could help identify provider‐level barriers to family communication and potentially inform improvements in GHP training and patient support.

## AUTHOR CONTRIBUTIONS

MB was involved in study design, data extraction, analysis, and manuscript writing. ÁM, CS, and MP contributed to study design, data analysis, manuscript writing, and editing. SFD and ÁM contributed to data extraction. All authors critically edited the manuscript.

## CONFLICT OF INTEREST STATEMENT

All the authors declare that they have no conflict of interest.

## ETHICS STATEMENT

Human and animal studies, and informed consent: Ethical approval was not required as only publicly available literature was used. No experiments were performed on human or animal subjects for this article.

## Supporting information


Table S1.

Table S2.

Table S3.

Table S4.

Table S5.

Table S6.

Table S7.

## Data Availability

All relevant data that support the findings of this study are within the paper and its supplemental files. Any additional information is available from the corresponding author upon request.

## Footnotes

^1^
An interactive treemap describing GHP's practices to address family communication is available at: https://public.flourish.studio/visualisation/22912904/.
