# Supplementary material for: Addressing family communication in genetic counseling: A scoping review of process studies
Source: J Genet Couns. 2025 Aug 13;34(4):e70067. doi: 10.1002/jgc4.70067 (PMC12345395; doi:10.1002/jgc4.70067)
Supplement: Supplementary file 2 — Table S2. [file JGC4-34-0-s006.docx]

*Table S2. Search terms and strategy*

| Search terms used in all databases | Database | Search strategy | Results |
| --- | --- | --- | --- |
| ("communicat*" OR "disclos*" OR "shar*" OR "inform*"OR "dissemin*")  AND  ("family" OR "relatives" OR "offspring" OR "parent*" OR "sibling*")  AND  ("genetic counsel*" OR "clinical genetics” OR "genetic services" OR “health professional*”)  AND  ("genetic info*" OR "genetic risk" OR "genetic results" OR "genetic variant*" OR "genetic disease" OR "hereditary disease")  AND  ("process*" OR "genetic counseling practice*" OR "health professional interaction*" OR "genetic counseling technique*" OR "professional patient relation*" OR "professional family relation*") | PsycINFO | TI ( ("communicat*" OR "disclos*" OR "shar*" OR "inform*"OR "dissemin*") AND ("family" OR "relatives" OR "offspring" OR ("parent*")) OR ("sibling*"))) AND ("genetic counsel*" OR "clinical genetics” OR "genetic services" OR “health professional*”) AND ("genetic info*" OR "genetic risk" OR "genetic results" OR "genetic variant*" OR "genetic disease" OR "hereditary disease") AND ("process*" OR "genetic counseling practice*" OR "health professional interaction*" OR "genetic counseling technique*" OR "Professional Patient Relation*"OR "Professional Family Relation*") ) OR AB ( ("communicat*" OR "disclos*" OR "shar*" OR "inform*"OR "dissemin*") AND ("family" OR "relatives" OR "offspring" OR ("parent*")) OR ("sibling*"))) AND ("genetic counsel*" OR "clinical genetics” OR "genetic services" OR “health professional*”) AND ("genetic info*" OR "genetic risk" OR "genetic results" OR "genetic variant*" OR "genetic disease" OR "hereditary disease") AND ("process*" OR "genetic counseling practice*" OR "health professional interaction*" OR "genetic counseling technique*" OR "Professional Patient Relation*"OR "Professional Family Relation*") ) NOT (review of literature or literature review or meta-analysis or systematic review)  Filters:  Year of Publication: 1997-2023  Scientific Journals (Peer-Reviewed)  Population Group: Human  Document Type: Journal Article  Exclude Book Reviews  Publication Type: All Journals  Restrict by Language:  - Spanish; Castilian  - English  Search Modes - Boolean/Phrase | 73 |
|  | PubMed | ((((((((((communicat*[Title/Abstract] OR "disclos*"[Title/Abstract] OR "shar*"[Title/Abstract] OR "inform*"[Title/Abstract] OR "dissemin*"[Title/Abstract]) AND "Family"[Title/Abstract] OR "relatives"[Title/Abstract] OR "offspring"[Title/Abstract] OR "parent*"[Title/Abstract] OR "sibling*"[Title/Abstract]) AND "genetic counsel*"[Title/Abstract] OR "clinical genetics"[Title/Abstract] OR "genetic services"[Title/Abstract] OR "health professional*"[Title/Abstract]) AND "genetic info*"[Title/Abstract] OR "genetic risk"[Title/Abstract] OR "genetic results"[Title/Abstract] OR "genetic variant*"[Title/Abstract] OR "genetic disease"[Title/Abstract] OR "hereditary disease"[Title/Abstract]) AND "process*"[Title/Abstract]) OR "genetic counseling practice"[Title/Abstract] OR "health professional interaction*"[Title/Abstract] OR "genetic counseling technique*"[Title/Abstract] OR "professional patient relation*"[Title/Abstract] OR "professional family relation*"[Title/Abstract]) AND ("english"[Language] OR "portuguese"[Language] OR "spanish"[Language]) NOT "systematic review"[Publication Type] NOT "review"[Publication Type] NOT "meta-analysis"[Publication Type]) AND 1997/01/01:2023/12/31[Date - Publication] AND "human*"[MeSH Terms] AND "genetic counsel*"[MeSH Terms] | 247 |
|  | Scopus | TITLE-ABS ("communicat*") OR TITLE-ABS ("disclos*") OR TITLE-ABS ("shar*") OR TITLE-ABS ("inform*") OR TITLE-ABS ("dissemin*") AND TITLE-ABS ("family") OR TITLE-ABS ("relatives") OR TITLE-ABS ("offspring") OR TITLE-ABS ("parent*") OR TITLE-ABS ("sibling*") AND TITLE-ABS ("genetic counsel*") OR TITLE-ABS ("clinical genetics") OR TITLE-ABS ("genetic services") OR TITLE-ABS ("health professional*") OR TITLE-ABS ("genetic info*") OR TITLE-ABS ("genetic risk") OR TITLE-ABS ("genetic results") OR TITLE-ABS ("genetic variants") OR TITLE-ABS ("genetic disease") OR TITLE-ABS ("hereditary disease") AND TITLE-ABS ("process*") OR TITLE-ABS ("genetic counseling practice*") OR TITLE-ABS ("health professional interaction*") OR TITLE-ABS ("genetic counseling technique*") OR TITLE-ABS ("Professional Patient Relation*") OR TITLE-ABS ("Professional Family Relation*") AND PUBYEAR > 1996 AND PUBYEAR < 2024 AND INDEXTERMS ("human*") AND INDEXTERMS ("Genetic counsel*") AND (EXCLUDE (DOCTYPE, "re") OR EXCLUDE (DOCTYPE, "cp") OR EXCLUDE (DOCTYPE, "ch")) AND (LIMIT-TO (LANGUAGE, "English") OR LIMIT-TO (LANGUAGE, "Spanish") OR LIMIT-TO (LANGUAGE, "French")) | 338 |
|  | Web of Science | (((((((((((((((((((((((((((TI=("communicat*")) OR TI=("disclos*")) OR TI=("shar*")) OR TI=("inform*")) OR TI=("dissemin*")) AND TI=("family")) OR TI=("relatives" )) OR TI=("offspring")) OR TI=("parent*")) OR TI=("sibling*")) AND TI=("genetic counsel*")) OR  TI=( "clinical genetics" )) OR TI=("genetic services")) OR TI=("health professional*")) OR TI=( "genetic info*")) OR TI=( "genetic risk")) OR TI=( "genetic results" )) OR TI=("genetic variant*")) OR TI=("genetic disease")) OR TI=("hereditary disease")) AND TI=("process*")) OR TI=("genetic counseling practice*")) OR TI=("health professional interaction*")) OR TI=("genetic counseling technique*")) OR TI=("Professional Patient Relation*")) OR TI=("Professional Family Relation*")) AND MH=("human*")) AND MH=("Genetic Counsel*")  OR  (((((((((((((((((((((((((((AB=("communicat*")) OR AB=("disclos*")) OR AB=("shar*")) OR AB=("inform*")) ORAB=("dissemin*")) AND AB=("family")) OR AB=("relatives" )) OR AB=("offspring")) OR AB=("parent*")) OR AB=("sibling*")) AND AB=("genetic counsel*")) OR AB=( "clinical genetics" )) OR AB=("genetic services")) OR AB=("health professional*")) OR AB=( "genetic info*")) OR AB=( "genetic risk")) OR AB=( "genetic results" )) OR AB=("genetic variant*")) OR AB=("genetic disease")) OR AB=("hereditary disease")) AND AB=( "process*")) OR AB=("genetic counseling practice*")) OR AB=("health professional interaction*")) OR AB=("genetic counseling technique*")) OR AB=("Professional Patient Relation*")) OR AB=("Professional Family Relation*")) AND MH=("human*")) AND MH=("Genetic Counsel*")  Filters:  French or English or Portuguese or Spanish (Languages)  Review Article or Meeting or Editorial Material or Abstract or Reference Material (Exclude – Document Types)  1997/01/01:2023/12/31[Date - Publication] | 354 |
