# Supplementary material for: Addressing family communication in genetic counseling: A scoping review of process studies
Source: J Genet Couns. 2025 Aug 13;34(4):e70067. doi: 10.1002/jgc4.70067 (PMC12345395; doi:10.1002/jgc4.70067)
Supplement: Supplementary file 4 — Table S4. [file JGC4-34-0-s005.docx]

*Table S4. Frequencies and percentages of the characteristics of included studies*

| Characteristics | | n | % |
| --- | --- | --- | --- |
| *Countries* | | |  |
| Australia | | 6 | 29 |
| United States of America | | 5 | 24 |
| Canada | | 2 | 10 |
| France | | 2 | 10 |
| Australia and the United Kingdom | | 1 | 5 |
| Netherlands | | 1 | 5 |
| Portugal | | 1 | 5 |
| Singapore | | 1 | 5 |
| Switzerland | | 1 | 5 |
| Not specified ª | | 1 | 5 |
| *Genetic Conditions* | | | |
| Multiple conditions | | 11 | 52 |
| One type of condition | | 10 | 48 |
| Type* | | | |
| Hereditary cancers* | | 13 | 62 |
|  | Hereditary breast and ovarian cancer | 9 | 43 |
|  | Hereditary colorectal cancer ^b^ | 4 | 19 |
|  | Hereditary diffuse gastric cancer | 1 | 5 |
|  | Unspecified hereditary cancers | 2 | 10 |
| Chromosomal translocations | | 3 | 14 |
| Cystic fibrosis | | 2 | 10 |
| 22q11.2 deletion syndrome | | 2 | 10 |
| Huntington's disease | | 2 | 10 |
| Hereditary cardiac conditions* | | 2 | 10 |
|  | Hypertrophic cardiomyopathy | 2 | 10 |
|  | Dilated cardiomyopathy | 1 | 5 |
| Amyotrophic lateral sclerosis | | 1 | 5 |
| Frontotemporal dementia | | 1 | 5 |
| Hemophilia | | 1 | 5 |
| Hemorrhagic telangiectasia | | 1 | 5 |
| Hereditary hemochromatosis | | 1 | 5 |
| KBG syndrome | | 1 | 5 |
| Legius syndrome | | 1 | 5 |
| Marfan syndrome | | 1 | 5 |
| Neurofibromatosis | | 1 | 5 |
| Phenylketonuria | | 1 | 5 |
| Sickle cell disease | | 1 | 5 |
| Thalassemia | | 1 | 5 |
| Von Willebrand disease | | 1 | 5 |
| XYY syndrome | | 1 | 5 |
| Not specified | | 1 | 5 |
| *GHP and other professionals providing care* | | | |
| Multidisciplinary care | | 15 | 71 |
| Intradisciplinary care | | 5 | 24 |
| Not specified | | 1 | 5 |
| Professionals* | | | |
| Unspecified professionals | | 9 | 43 |
|  | Unspecified medical professionals | 6 | 29 |
|  | Unspecified GHP | 2 | 10 |
|  | Unspecified mental health professionals | 1 | 5 |
| Genetic counselors | | 16 | 76 |
| Medical geneticists | | 10 | 48 |
| Nurses | | 4 | 19 |
| Oncologists | | 3 | 14 |
| Obstetricians and gynecologists | | 2 | 10 |
| Psychiatrists | | 2 | 10 |
| Social workers | | 1 | 5 |
| Clinical researchers | | 1 | 5 |
| Fertility specialists | | 1 | 5 |
| Gastroenterologists | | 1 | 5 |
| General practitioners | | 1 | 5 |
| Medical assistants | | 1 | 5 |
| Neurologists | | 1 | 5 |
| Nutritionists | | 1 | 5 |
| Psychologists | | 1 | 5 |
| *Authors’ background* | | | |
| Multidisciplinary research teams | | 16 | 76 |
| Intradisciplinary research team | | 5 | 24 |
| Areas* | | | |
|  | GC | 14 | 67 |
|  | Clinical genetics | 11 | 52 |
|  | Psychology | 6 | 29 |
|  | Bioethics | 5 | 24 |
|  | Health behavior | 4 | 19 |
|  | Health communication | 4 | 19 |
|  | Healthcare, public, and population health research | 4 | 19 |
|  | Oncology | 3 | 14 |
|  | Epidemiology | 2 | 10 |
|  | Health sociology | 2 | 10 |
|  | Nursing | 2 | 10 |
|  | Family health | 1 | 5 |
|  | Medical anthropology | 1 | 5 |
|  | Psychiatry | 1 | 5 |
| *Methodology* | | | |
| Qualitative | | 15 | 71 |
| Mixed methods | | 5 | 24 |
| Quantitative | | 1 | 5 |
| Retrospective | | 19 | 90 |
| Prospective | | 2 | 10 |
| Data collection | | | |
| Single method | | 15 | 71 |
|  | Semi-structured interviews | 9 | 43 |
|  | Surveys | 2 | 10 |
|  | Clinical records | 2 | 10 |
|  | Video-recordings of consultations | 1 | 5 |
|  | Ethnographic survey | 1 | 5 |
| Multi-method | | 6 | 29 |
|  | Semi-structured interviews and focus groups | 2 | 10 |
|  | Semi-structured interviews and surveys | 2 | 10 |
|  | Semi-structured interviews, focus groups and surveys | 2 | 10 |
| Data analysis | | | |
| Single method | | 16 | 76 |
|  | Thematic analysis | 6 | 29 |
|  | Grounded theory | 5 | 24 |
|  | Content analysis | 3 | 14 |
|  | Interpretative description | 1 | 5 |
|  | Statistical analysis | 1 | 5 |
| Multi-method | | 5 | 24 |
|  | Content analysis and statistical analysis | 3 | 14 |
|  | Thematic analysis and statistical analysis | 2 | 10 |
| *Participants* | | | |
| GHP and other professionals | | 8 | 38 |
| Patients | | 6 | 29 |
| Patients, GHP and other professionals | | 4 | 19 |
| Patients and relatives | | 2 | 10 |
| Relatives | | 1 | 5 |
| *Note.* Number of studies = 21, GC = genetic counseling; GHP = genetic health professionals  *Due to several studies including more than one type of genetic condition, and multidisciplinary care and research teams, percentages exceed 100% when totaled due to overlap.  ^a^ Countries from Africa, Australasia, Europe, Middle East, North and South America were included.  ^b^ Hereditary colorectal cancers included Lynch syndrome and familial adenomatous polyposis. | | | |
