# Supplementary material for: Addressing family communication in genetic counseling: A scoping review of process studies
Source: J Genet Couns. 2025 Aug 13;34(4):e70067. doi: 10.1002/jgc4.70067 (PMC12345395; doi:10.1002/jgc4.70067)
Supplement: Supplementary file 5 — Table S5. [file JGC4-34-0-s007.docx]

*Table S5. Patient suggestions of practices to support family communication*

| Author and year | Patient suggestions of practices to support family communication |
| --- | --- |
| Brown et al., 2021 | - Letters provided to patients should be personalized, address the psychological impact of a possible diagnosis, provide tailored resources and contacts for the family, and be sent via mail and email |
| Cook et al., 2022 | - Need for ongoing, personalized, and practical support (educational videos roleplaying family communication, local resources, next steps), and more directive guidance from GHP |
| Crook et al., 2022 | - Clarify familial implications of results - Provide information on local resources for patients and relatives to access testing - Provide personalized, ongoing support for the patient and their relatives to assist with family communication |
| Gaff et al., 2005 | - Discuss with patients how to share information with relatives - Provide additional communication aids (personalized letters, a list of at-risk relatives, and videos) |
| Hudson et al., 2019 | - Provide information and materials that would help relatives understand the condition, the importance of genetic testing, and how to access it (contacts of healthcare professionals) |
| Lafrenière et al., 2013 | - Further prompting, preparation (correct terminology), and support (communication aids) for family disclosure |
| Mendes & Sousa, 2012 | - GHP should have a proactive role in communicating genetic information to at-risk relatives |
| Pedrazzani et al., 2022 | - GHP should have a proactive role in communicating genetic information to at-risk relatives - GHP should be forthright when addressing the importance of family communication, and revisit the topic at follow-up |
