# Supplementary material for: Addressing family communication in genetic counseling: A scoping review of process studies
Source: J Genet Couns. 2025 Aug 13;34(4):e70067. doi: 10.1002/jgc4.70067 (PMC12345395; doi:10.1002/jgc4.70067)
Supplement: Supplementary file 6 — Table S6. [file JGC4-34-0-s001.docx]

| Author and year | Needs of GHP for further resources |
| --- | --- |
| Forrest et al., 2010 | - The main barrier to the provision of follow-up support in family communication of genetic risk identified by GHP is the lack of time, administrative and clinical support |
| Gorrie et al., 2018 | - Key informants, including GHP, considered there was a need for further training for healthcare professionals about genetic conditions and the availability of screening tests, to better support carriers during family communication of genetic risk - Key informants felt it was needed provide carriers with additional follow-up support, and relevant information for their reference during family communication - Of researcher-suggested materials to support family communication follow-up letters were considered redundant by informants - Mailed information packs, and online resources (including information about the condition, genetic testing, at-risk relatives and information on communicating with relatives) were considered useful by informants and patients - Of the researcher-suggested support, follow-up appointments were considered useful by informants and patients - Involving support groups was considered challenging to coordinate with GC by informants - GHP directly contacting relatives through letters was considered useful by informants, but not by patients |
| Young, Butow, Tucker, et al., 2019 | - Need for strategies to probe patients about family communication (tools to inform the types of questions you might ask to explore family dynamics) - Need for further training in behavioral change techniques and theoretical knowledge of family systems and communication |
| Young et al., 2020 | - Some GHP feel that the resources available to assist with family communication are often too generic or not user-friendly - Some GHP reported that supporting families during communication and family group consultations are difficult to do in the current public health model and would require more resources (e.g. time) |

*Table S6. Reported needs of GHP for further resources to address family communication*
