# Supplementary material for: Addressing family communication in genetic counseling: A scoping review of process studies
Source: J Genet Couns. 2025 Aug 13;34(4):e70067. doi: 10.1002/jgc4.70067 (PMC12345395; doi:10.1002/jgc4.70067)
Supplement: Supplementary file 7 — Table S7. [file JGC4-34-0-s002.docx]

*Table S7. Contextual influences on how family communication is addressed by GHP*

| Author and year | Contextual influences on how family communication is addressed by GHP |
| --- | --- |
| Brown et al., 2021 | - Letter format (physical or online) influenced how it was used during family communication |
| Crook et al., 2022 | - Practices to address family communication varied between GHP, with some creating communication barriers (e.g. only giving a copy of lab results to other healthcare professionals) |
| Gaff et al., 2005 | - Family communication is discussed since the first GC appointment |
| Lafrenière et al., 2013 | - Practices to address family communication varied between GHP. - Not all GHP highlighted the importance of family communication with patients or encouraged them to share genetic information with relatives. |
| Pedrazzani et al., 2022 | - Family communication is usually not addressed after the post-testing consultation - The depth in which family communication is discussed varied between GHP, with some being more superficial and abstract, while others were more detailed - Lack of coordination from multidisciplinary care teams - Additional (follow-up) support was only provided if patients requested it |
| Clarke et al., 2005 | - Family communication is addressed throughout GC - GHP prefer patient-mediated family disclosure over direct communication with relatives |
| d’Audiffret Van Haecke & de Montgolfier, 2016 | - GHP struggle with discussing family risk before knowing test results, with some having more structured consultations while others adapted consultations to each patient - Family-related discussions begin by mapping genetic genealogy in pre-test and continue throughout GC, with supportive documents and consent forms that mention family communication |
| Derbez et al., 2017 | - Family communication is addressed before (family request forms), throughout (GHP and consent forms), and after GC - GHP prefer patient-mediated family disclosure over direct communication with relatives |
| Forbes Shepherd et al., 2017 | - A relational approach is used solely for those accepting to be tested - The covert stage is the most used in GC practice - The overt stage is used when there is a reluctance from patients to disclose information to at-risk relatives - The authoritative stage is only used when there is a refusal to disclose information to relatives - Some GHP used the consent form to revisit discussions on familial implications of testing (relational consent) - Some GHP omit the option of non-disclosure of genetic information to the family |
| Forrest et al., 2010 | - There is high consistency among GHP in the type of practices used to address family communication, even though the frequency with which these practices were used varied considerably - Most GHP prefer patient-mediated family disclosure over direct communication with relatives - A minority of GHP believe patients do not require support after GC, as such getting it is sometimes reliant on seeking assistance from GHP |
| Gallo et al., 2010 | - Only genetic counselors and social workers were involved in addressing family communication - While genetic counselors suggested parents share information with at-risk relatives, some of the other professionals viewed it as a personal choice - Easier to discuss family communication in treatable or recessive conditions and in appropriate physical settings |
| Gorrie et al., 2018 | - Telehealth follow-up was considered more useful than direct contact from GHP to relatives and in-person or letter follow-ups, with the suggested timing being within a couple of weeks of result disclosure |
| Makhnoon et al., 2021 | - Practices to address family communication varied between GHP - Specific familial genetic risk messaging was more frequent for high-risk variants and in group appointments |
| Stol et al., 2010 | - Most GHP avoid direct disclosure to relatives - Most GHP avoid being directive with patients mentioning relatives’ right not to know, legal restrictions, patient responsibility to inform relatives, and lack of resources to directly contact relatives |
| Young, Butow, Tucker et al., 2019 | - GHP cue patients to begin thinking early in GC about family communication - Pre-counseling phone calls (to discuss family dynamics) are used to adapt GC appointments (setting, family or individual appointments, how to address family disclosure with each patient) |
| Young et al., 2020 | - Family communication is addressed since the first appointment - Practices to address family communication vary between GHP with some adapting their practices based on the condition, type of genetic test (diagnostic or pre-symptomatic testing) and available resources in their GC service |
